# Urinary Colorimetric Sensor Array and Algorithm to Distinguish Kawasaki Disease from Other Febrile Illnesses

**DOI:** 10.1371/journal.pone.0146733

**Published:** 2016-02-09

**Authors:** Zhen Li, Zhou Tan, Shiying Hao, Bo Jin, Xiaohong Deng, Guang Hu, Xiaodan Liu, Jie Zhang, Hua Jin, Min Huang, John T. Kanegaye, Adriana H. Tremoulet, Jane C. Burns, Jianmin Wu, Harvey J. Cohen, Xuefeng B. Ling, Emergency Medicine Kawasaki Disease Research Group

**Affiliations:** 1 Institution of Microanalytical System, Zhejiang University, Hangzhou, Zhejiang, China; 2 Department of Surgery, Stanford University, Stanford, California, United States of America; 3 Shanghai Children's Hospital, Shanghai Jiao Tong University, Shanghai, China; 4 Department of Pediatrics, University of California San Diego, La Jolla, California, United States of America; 5 Rady Children’s Hospital San Diego, San Diego, California, United States of America; 6 Department of Pediatrics, Stanford University, Stanford, California, United States of America; University of Houston, UNITED STATES

## Abstract

**Objectives:**

Kawasaki disease (KD) is an acute pediatric vasculitis of infants and young children with unknown etiology and no specific laboratory-based test to identify. A specific molecular diagnostic test is urgently needed to support the clinical decision of proper medical intervention, preventing subsequent complications of coronary artery aneurysms. We used a simple and low-cost colorimetric sensor array to address the lack of a specific diagnostic test to differentiate KD from febrile control (FC) patients with similar rash/fever illnesses.

**Study Design:**

Demographic and clinical data were prospectively collected for subjects with KD and FCs under standard protocol. After screening using a genetic algorithm, eleven compounds including metalloporphyrins, pH indicators, redox indicators and solvatochromic dye categories, were selected from our chromatic compound library (n = 190) to construct a colorimetric sensor array for diagnosing KD. Quantitative color difference analysis led to a decision-tree-based KD diagnostic algorithm.

**Results:**

This KD sensing array allowed the identification of 94% of KD subjects (receiver operating characteristic [ROC] area under the curve [AUC] 0.981) in the training set (33 KD, 33 FC) and 94% of KD subjects (ROC AUC: 0.873) in the testing set (16 KD, 17 FC). Color difference maps reconstructed from the digital images of the sensing compounds demonstrated distinctive patterns differentiating KD from FC patients.

**Conclusions:**

The colorimetric sensor array, composed of common used chemical compounds, is an easily accessible, low-cost method to realize the discrimination of subjects with KD from other febrile illness.

## Introduction

Kawasaki disease (KD), an acute pediatric vasculitis, has become the leading cause of acquired heart disease in children in the United States [1]. If not treated promptly, KD patients may develop coronary artery dilatation or aneurysms, which can be largely prevented by early administration of intravenous immunoglobulin (IVIG) [2]. The diagnosis is difficult, given the overlap in clinical presentation with other febrile illnesses in childhood. Therefore, the development of a low-cost, rapid, and practical test for the diagnosis of KD at the point of care is a high priority.

Colorimetric array-based sensing is a powerful tool for the quantitative detection of chemically diverse analytes by recognition of a distinctive pattern with multiple sensing compounds [3–5]. The most commonly used sensing compound classes for a colorimetric sensor are common chemical reagents, e.g. metalloporphyrins, Brønsted acid/base dyes, solvatochromic dyes, and redox indicators. Metalloporphyrins are a natural choice of a Lewis acid/base dye that can be used for recognition of analytes with Lewis acid/base capabilities. They have large spectral shifts upon ligand binding, providing ligand differentiation based on metal-selective coordination and cross-respond to varying degrees to a wide variety of different analytes. Brønsted acid/base dyes respond not only to the change of proton acidity or basicity but also to the chemical reaction with the solvent environment. Solvatochromic dyes change color in response to changes in the polarity of their environment, especially the solvent environment polarity. Redox indicators, including metal-organic complexes and true organic redox systems undergo a definite and reversible color change between oxidized and reduced forms. Sensor specificity is achieved through quantitative digital imaging and analysis of the composite color difference map. Such cross-reactive and unique composite response sensor arrays have been studied for the identification of toxic industrial gases and vapors [5–8] and organic compound analytes in aqueous liquids [4, 9]. It had also been used for clinical specimens, such as cancer diagnosis based on exhaled gases [10]. The colorimetric array sensing compounds can be easily procured at low cost, and the sensing array can be reduced to a microarray format with high-throughput printing technology.

We present the first attempt to use this colorimetric sensor array for urine-based diagnosis.

## Materials and Methods

### Ethics

This study was approved by the Institutional Review Boards of the University of California, San Diego (UCSD) and Stanford University. Signed informed consent was obtained from all the subjects, and child or adolescent assent was obtained as appropriate. We received signed consent from the next of kin, caretakers, or guardians on behalf of the minors/children enrolled in our study.

### Patient demographics and samples

The diagnosis of KD was validated by one of two KD expert clinicians (AHT or JCB) at the KD Research Center in UCSD according to an established protocol with standardized, prospective data collection. Inclusion criteria for KD subjects were based on the American Heart Association Guidelines [11]. Febrile controls (FC) were sex- and age-matched children who presented to the Emergency Department of Rady Children’s Hospital in San Diego with fever for ≥3 days and at least one of the clinical signs of KD: rash, conjunctival injection, oral mucosa changes, extremity changes, and enlarged cervical lymph node. Each FC’s diagnosis was adjudicated by a chart review by two expert clinicians after all culture and laboratory data were available. Urine samples were collected by standard methods chosen by the treating clinician and processed as previously described [12]. There were 49 sex- and age-matched KD and 50 FC subjects in this study (Tables 1 and 2), in which 2 detailed clinical information of FC subjects are currently unavailable.

**Table 1:** Demographics analysis. Clinical characteristics of acute KD and age/gender matched FC subjects.

	Training (FC, n = 29; KD, n = 33)			Testing (FC, n = 17; KD, n = 16)		
	FC	KD	*P*	FC	KD	*P*
**Age (month)**			0.0411			0.787
**Median (IQR)**	67.1 (49.4,93.3)	52.6 (24.7,65.3)		42.3 (26.6,100.7)	52.8 (34.1,66.8)	
**Sex, n(%)**			0.6052			0.398
**Female**	18 (58.1)	16 (48.5)		15 (88.2)	12 (75)	
**Race, n(%)**			<0.0013			0.286
**African American**	0 (0)	1 (3.0)		0 (0)	0 (0)	
**Asian**	1 (3.2)	5 (15.2)		1 (5.9)	1 (6.2)	
**Caucasian**	16 (51.6)	5 (15.2)		4 (23.5)	2 (12.5)	
**Hispanic**	8 (25.8)	12 (36.4)		8 (47.1)	6 (37.5)	
**Mixed**	5 (16.2)	9 (27.3)		4 (23.5)	7 (43.8))	
**Native American**	1 (3.2)	0 (0)		0 (0)	0 (0)	

**Table 2:** Diagnosis of FCs. Diagnoses of febrile control subjects in the training and testing cohorts.

Diagnosis of FCs	Training	Testing
	(FC, n = 33)	(FC, n = 17)
**Bacterial infections**		
Scarlet fever	1	0
Staphylococcal scalded skin syndrome	1	1
pharyngitis	1	0
Lymphadenitis	2	0
Total (%)	5 (15.2)	1 (5.9)
**Viral infections**		
Adenovirus	1	4
Viral syndrome	15	4
Influenza virus	3	0
Enterovirus	2	0
Others	4	5
Total (%)	25(75.8)	13(76.4)
**Both bacterial and viral infection (%)**	1(3.0)	1(5.9)
**Other inflammatory processes (%)**	2(6.0)	2(11.8)

### Colorimetric sensor array assembling

A total of 190 candidate compounds, (S1 Table) including metalloporphyrins, pH indicators, redox indicators and solvatochromic dye categories were procured from Sigma-Aldrich or Fisher Scientific Inc. Hydrophobic colorimetric dyes were dissolved in DMSO, and water-soluble compounds were directly dissolved in deionized water. The colorimetric sensor array was in a 384-well format with 10 μL stock compound solution thoroughly mixed with 10 μL of urine per well. Saline (0.9% NaCl, w/w) was used as a blank.

### Digital imaging analysis and normalization of compound readout

A flatbed scanner was used for image collection with the upper side of the array facing toward the scanner. Difference color maps were obtained as previously described [13] by taking the difference of the red, green and blue (RGB) values from every colorant spot from the ‘blank’ and ‘sample’ images. For each urine sample, the image analysis led to a color-difference vector of 3×N compound features, where N was the total number of array sensing compounds. To improve visualization of the color changes, expanded RGB color difference patterns were used.

### Urine sampling and dilution effect normalization

To minimize potential bias or confounding factors, it is essential that urine samples are collected and handled in standardized ways [14]. Urine samples were collected as previously described [12]. Urine profiling analyses suffer two major different origins of variance: analytical issues due to platform introduced variances; biological issues including dilution of urine by different hydration states of the urine donors [14]. To correct the urine dilution effect, the compound readout was normalized to urinary creatinine concentration [14].

### KD diagnostic algorithm development

A genetic algorithm (GA) [15] was applied to select compounds significant for KD/FC discrimination. GA’s “evolution process” [16] was repeated to construct an optimal subset of sensing compounds and develop a decision-tree based [17] KD diagnostic algorithm with the maximum receiver operating characteristic (ROC) area under curve (AUC) value. The KD diagnostic algorithm assigned a probabilistic score for each sample.

### Statistical analysis

Patient demographic data were analyzed using the Epidemiological calculator (R epicalc package). Student’s t test was performed to calculate *P* values for continuous variables, and Fisher’s exact test was used for comparative analysis of categorical variables. Hypothesis testing was performed using the Mann–Whitney U -test (two tailed).

### Sensing compound literature association analysis

The literature mining was performed to uncover published associations between our 11 sensing compounds and biological functions/processes using R RISmed package.

## Results

### Demographics

The study was approved by the Institutional Review Boards (IRBs) of the University of California, San Diego (UCSD) and Stanford University. We collected a total of 99 urine samples from consented KD and febrile control (FC) subjects following guardian consent and child or adolescent assent as appropriate. Patient characteristics and the distribution of diagnoses for the FCs were similar between the training and testing cohorts (Table 1). KD subjects in the training cohort were younger and more often Hispanic. FC diagnoses in both training and testing cohort were most commonly viral (~75%) and bacterial (~10%) infections (Table 2).

### Colorimetric sensor array and discriminant compound selection

**Fig 1:**
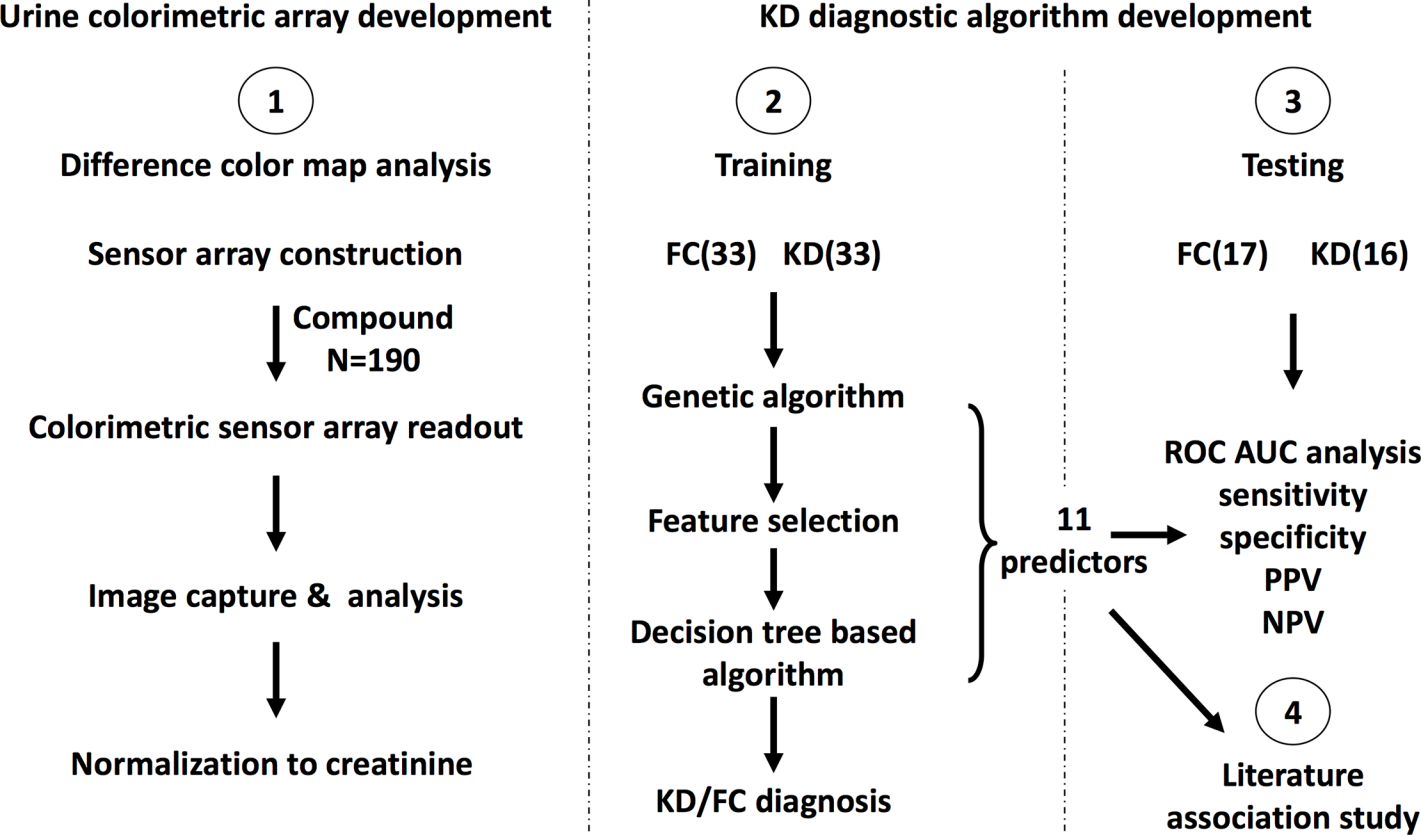
Experimental design to assemble a colorimetric urine sensor array to diagnose KD from FC subjects.

**Fig 2:**
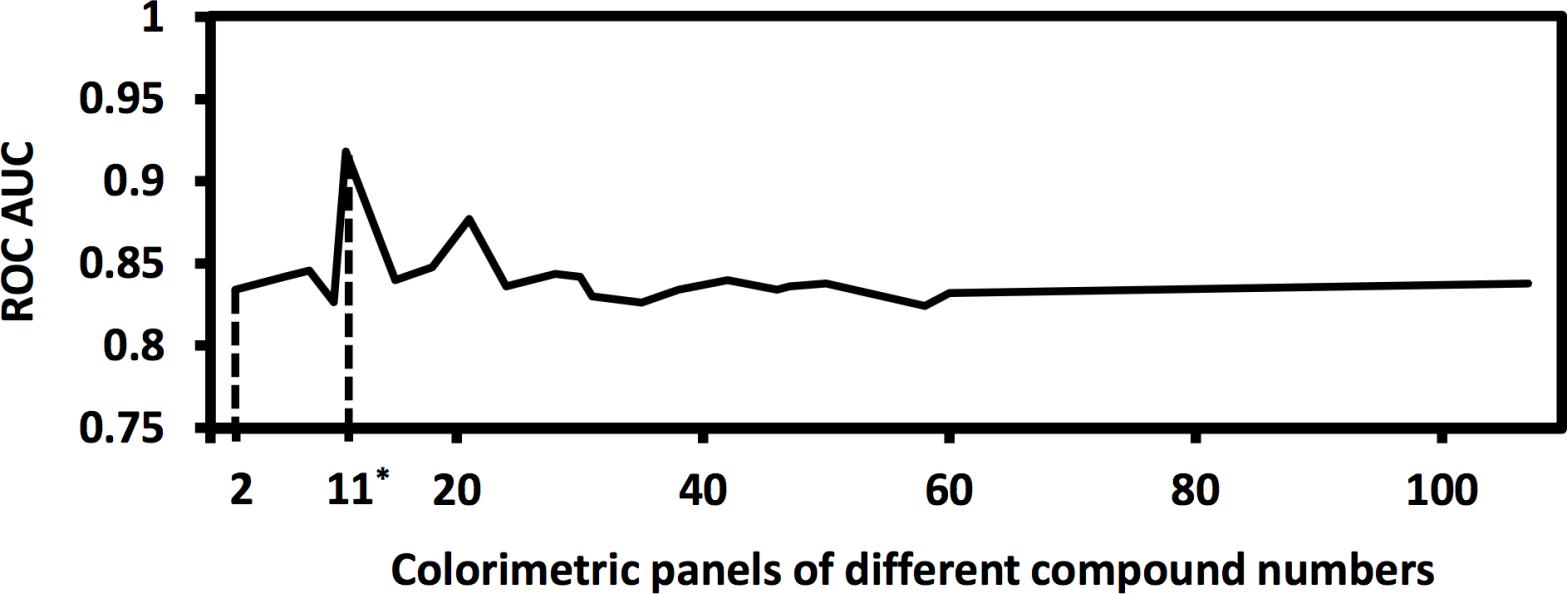
Predictor sensing compound selection. A genetic algorithm was applied for feature selection from total of 190 candidate compounds. 11 predictor sensing compounds were selected with the highest ROC AUC values achieved during GA’s “evolution process” process for the discrimination of KD from FC.

**Fig 3:**
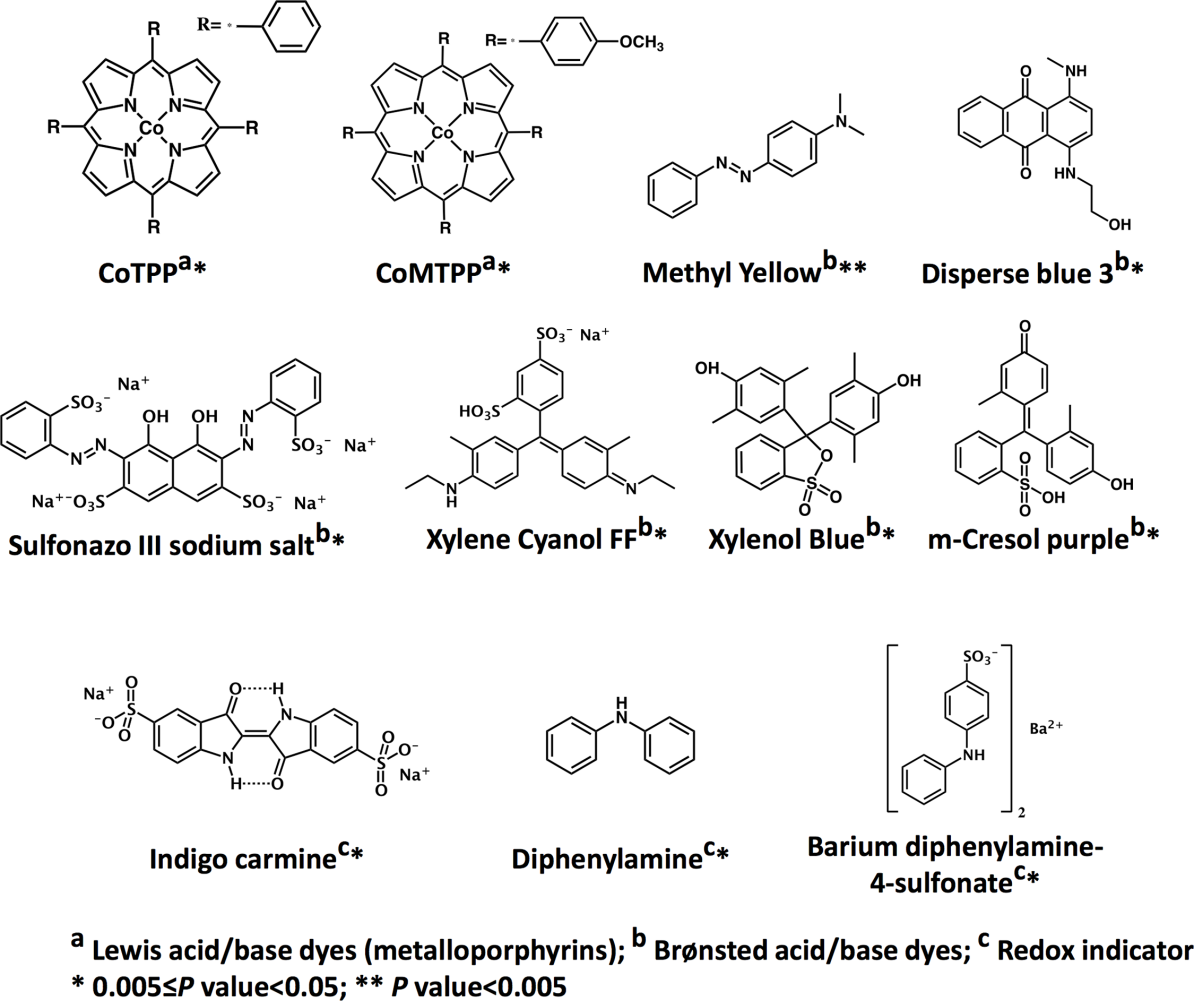
Predictor sensing compound structure. P value: Mann Whitney U test (two sided).

**Fig 4:**
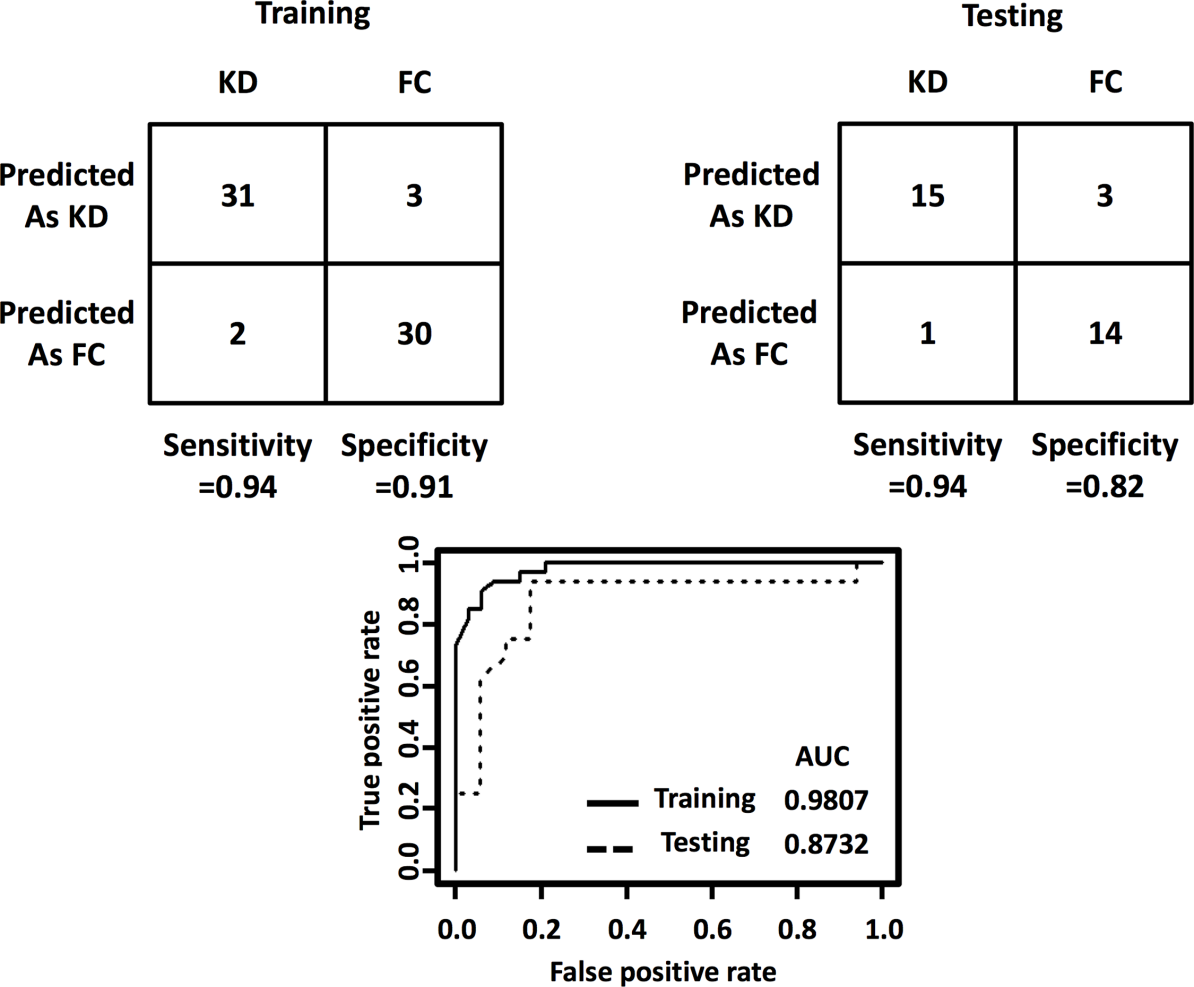
Performance of the KD diagnostic algorithm in discriminating KD from FC. Top, The 2 × 2 contingency tables that were used to calculate the percentage of classifications that agreed with the clinical diagnosis. Bottom, Receiver operating characteristic (ROC) curves for the ability of decision tree-based, algorithm-derived prediction scores to distinguish KD from FC in training and testing cohorts.

As outlined in Fig 1, the training cohort was used for predictor sensing compound discovery from the 190 candidate compounds (S1 Table) and supervised classification, and the testing cohort was used for class prediction of blinded urine samples. Panels of different combinations of the 190 sensing compounds were evaluated (Fig 2), which led to an optimal subset of 11 sensing compounds (Fig 3, S1 Fig). The derived decision-tree based KD diagnostic algorithm had sensitivity of 94%, specificity of 91%, and a receiver operating characteristic (ROC) area under curve (AUC) of 0.981 when applied to the training cohort (Fig 4).

### Testing of the KD diagnostic colorimetric array

**Fig 5:**
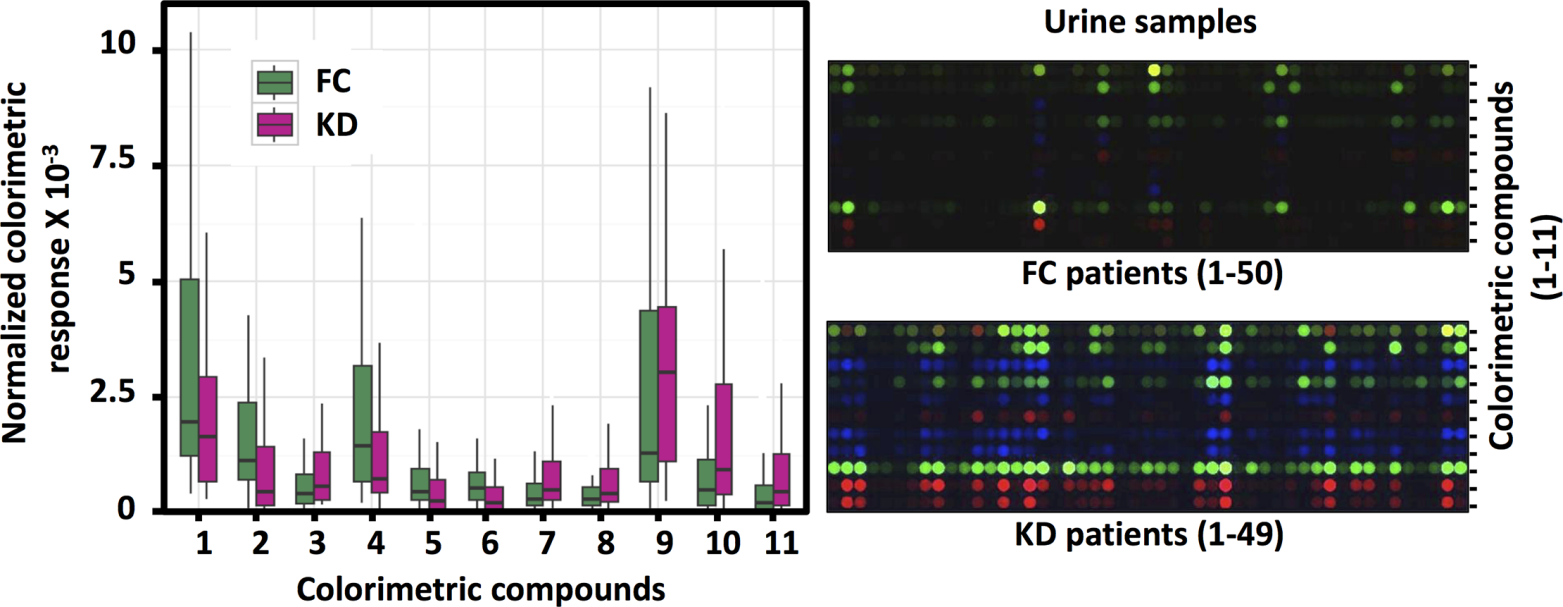
KD diagnosis with selected predictor sensing compounds. Left, box-whisker plot with creatinine-normalized colorimetric response to 11 selected compounds, where sea green represents FC and violet red represents KD. Right, colorimetric array difference color maps show distinct patterns differentiating KD and FC subjects.

When the 11-predictor colorimetric array was applied to the testing cohort, the decision-tree-based KD diagnostic algorithm classified these subjects with 94% sensitivity and 82% specificity. The ROC curve analysis using the testing cohort revealed an AUC of 0.873 (Fig 4). Consistent with these findings, colorimetric responses of these predictor compounds differed between KD and FC urine samples (Fig 5, left panel). The colorimetric array maps (Fig 5, right panel) revealed distinct patterns differentiating KD and FC subjects.

### Systematic association of sensing predictor compounds to biological function and process by literature mining

**Fig 6:**
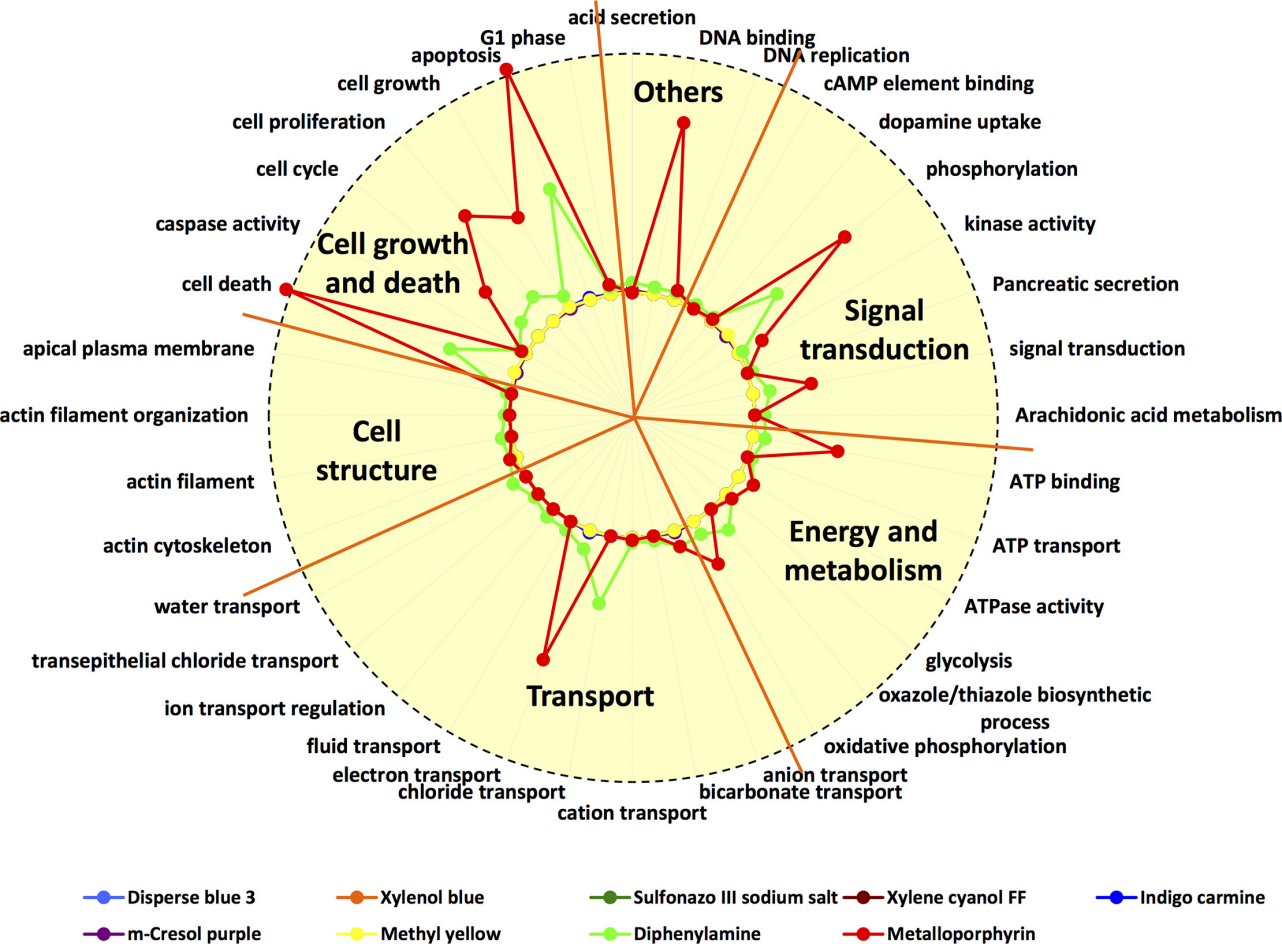
Radar plot summarizes the association of predictor compounds and underlying biological functions by literature mining. All functional annotations were classified into 6 different subtypes.

To explore the pathophysiology underlying the KD-specific compound sensing patterns, we used an unsupervised, systematic literature mining approach [18]. We computed associations between these predictor compounds and the gene ontology biological functions and processes within PubMed abstracts and titles, because the preferential co-occurrence of terms suggests an underlying mechanistic relationship. Six different types of biological functions, including cell growth and death, cell structure, transportation, energy and metabolism, signal transduction, and others (acid secretion, DNA binding, DNA replication) were found in association with the 11 compounds (Fig 6), suggesting that future characterization of the compound predictor compounds’ sensing mechanism may provide new clues for KD pathogenesis.

## Discussion

The KD etiology remains unknown, and currently, clinicians rely on clinical experience without the benefit of an objective molecular test at the point of care with desired sensitivity and specificity. The key problem in the diagnosis of KD is to improve the differentiation of children with KD from FCs that had other pediatric illness like adenovirus infection and scarlet fever. Thus, children with different kinds of rash-fever illnesses that mimic KD were used as controls in our study (Table 2). The diagnosis rests upon clinical criteria that are shared by other confounding pediatric febrile illnesses. In patients with fever lasting more than five days and two or three classic symptoms of Kawasaki disease, CRP and ESR should be measured. If those are high, measurement of serum albumin and serum transaminase levels, complete blood cell count, and urinalysis should be performed. However, clinical confusion from clinical criteria based upon AHA guidelines for diagnosing KD, including ESR [receiver operating characteristic (ROC) area under the curve (AUC): 0.689 for continuous ESR; 0.625 for elevated ESR≥40mm/hr], CRP (ROC AUC: 0.693 for continuous CRP; 0.619 for elevated CRP≥3mg/DL) and combination of elevated ESR or elevated CRP (ROC AUC: 0.568) as shown in S2 Fig, can lead to a missed or delayed diagnosis and timely treatment, resulting increased risk of coronary artery aneurysms. Our KD sensing array allowed the identification of 94% of KD subjects (ROC AUC: 0.981) in the training set and 94% of KD subjects (ROC AUC: 0.873) in the testing set.

Array-based sensing has emerged as a powerful tool for compound categorization by producing specificity through quantitative digital imaging and analysis of the composite color difference map. We are the first to explore the feasibility to apply colorimetric sensor array for disease diagnosis through the composite analysis of a large number of chemically diverse analytes in patient urine. Given that urine can be sampled frequently and non-invasively, urine based tests would be more applicable to the pediatric patients. Therefore, it would be ideal to test the platform with a pediatric disease having a good urine based test. However, since there is no objective diagnostics in Kawasaki disease, we aim to address this unmet medical need with an innovative application of the urine based sensor array technology, and the success of urine sensor assay in a different disease would not necessary warrant the effective clinical utility in Kawasaki disease.

Our literature association study explored the underlying mechanistic relationship between our sensing predictors and urine analyte patterns due to KD pathophysiology. Urine dipstick tests consist of strips with multiple reagent-impregnated pads which colorimetrically detect the presence of abnormal substances, e.g. heme, bilirubin, nitrite, and leukocyte esterase, in urine. The tests include profiling for the presence of urine albumin and/or Tamm-Horsfall protein with pH indicators, urine hemoglobin and myoglobin with chromogen tetramethylbenzidine, glucose with a chromogen, ketones with nitroferricyanide, bilirubin with diazonium salt, urobilinogen with p-dimethylaminobenzaldehide, nitrite with para-arsanilic acid or sulphanilamide and tetrahydrobenzoquinoline, and leukocytes with indolecarboxylic acid ester and diazonium salt for bacterial infection.

We believe that the high dimensionality of chemical sensing permits sensitive discrimination among very similar analytes and accurate profiling of urine over a wide range of analytes. This notion is supported by recent findings that Congo red can be used to identify atypical brain amyloidal aggregates in Alzheimer’s and prion disease, and misfolded proteins in urine from pregnant women with pre-eclampsia [19]. Biomarkers identified in KD urine including small molecular compounds (e.g. cysteinyl leukotrienes, nitrite or nitrate, and neopterin) [20–22], naturally occurring peptides [12], and proteins [23], might serve as the targets for the colorimetric sensor array. For example, the sensing compounds diphenylamine and barium diphenylamine-4-sulfonate, previously used for the colorimetric determination of nitrates [24, 25], form oxidation products in chromogenic reaction with urinary nitrate. Further work should clarify the nature of the distinct urine constituents that account for the diagnostic pattern in KD patients.

We set to develop compound sensory array urine test to discriminate KD from FC subjects. An age matched (P value 0.08) cohort, with FC subject age median (IQR) 54.8 (39.8,97.2) and KD subject age median (IQR) 52.6 (24.7,66.1) months, was constructed. Therefore, age alone has limited ability to differentiate the KD and FC subjects (ROC AUC values: 0.6049). However, such selection criteria caused our study cohort of recruited KDs’ age older than the KD median age of 2 years reported by hospital discharge data in the US in 2000 [26] (S3 Fig, top). To address whether age is a key factor influencing our compound sensor differentiating results, comparative analysis of different KD patient age groups’ sensor array results was performed. Mann-Whitney test showed that that there are no statistical differences between different age groups’ sensor differentiating results: age group cutoff at 2, 4, 6, 8, or 10 years (S3 Fig, bottom). Future prospective study will test KD and FC subjects of all ages in clinics.

Although a sensing compound panel was discovered to diagnose KD, there is still lack of estimated mechanism to map out the existing knowledge and gaps in understanding these KD sensing compounds. We believe that no single compound will be able to predict KD, and the future optimized sensor array would be a panel of compounds. Therefore, understanding the mechanisms of each compound sensing action can offer the best route to optimize the array’s KD/FC differentiating abilities. Future biochemical studies will be needed to characterize the compound binding entities in the urine matrix to identify all the pathophysiological pathways activated in this sensing condition.

**Fig 7:**
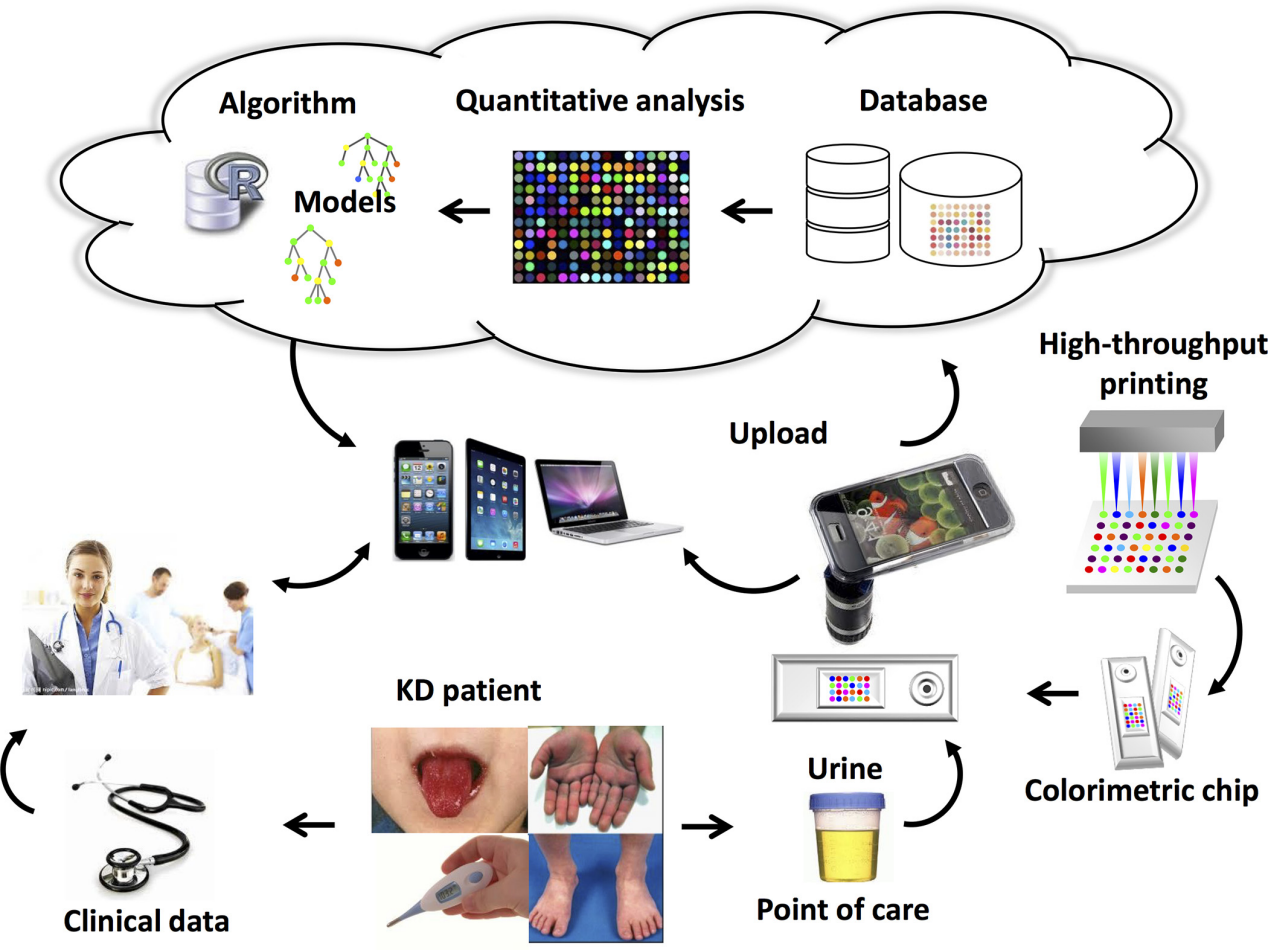
Schematic workflow of the future point of care colorimetric array for KD management.

In this study, we describe a colorimetric urine test that differentiated cohorts of KD and FC subjects with sufficient accuracy to be clinically useful. To our knowledge, this study is the first to apply colorimetric array-based sensing technology in a urine-based diagnostic test. Following larger scale validation of this prototype diagnostic test, this colorimetric array, formatted for single patient, would be appropriate for the emergency department or clinical setting (Fig 7).

## Supporting Information

S1 Fig Performance of each sensor compound in the urine assay to discriminate KD from FC subjects. (PDF)

S2 Fig Use of continuous or elevated ESR, CRP, or a combination of elevated ESR or elevated CRP to diagnose KD from FC subjects. (PDF)

S3 Fig Top: Age distribution among the KD subjects. Bottom: Comparison of the algorithm-derived prediction scores be-tween younger and older groups of KD subjects.
(PDF)

S1 Table The compound candidates used to construct colorimetric sensor array for KD discrimination. (XLSX)
