# Supplementary material for: Urinary Colorimetric Sensor Array and Algorithm to Distinguish Kawasaki Disease from Other Febrile Illnesses
Source: PLoS One. 2016 Feb 9;11(2):e0146733. doi: 10.1371/journal.pone.0146733 (PMC4747548; doi:10.1371/journal.pone.0146733)
Supplement: S1 Fig — (PDF) [file pone.0146733.s001.pdf]

Supplementary Figure 1. Performance of each sensor compound in the urine assay to discriminate KD from FC subjects.

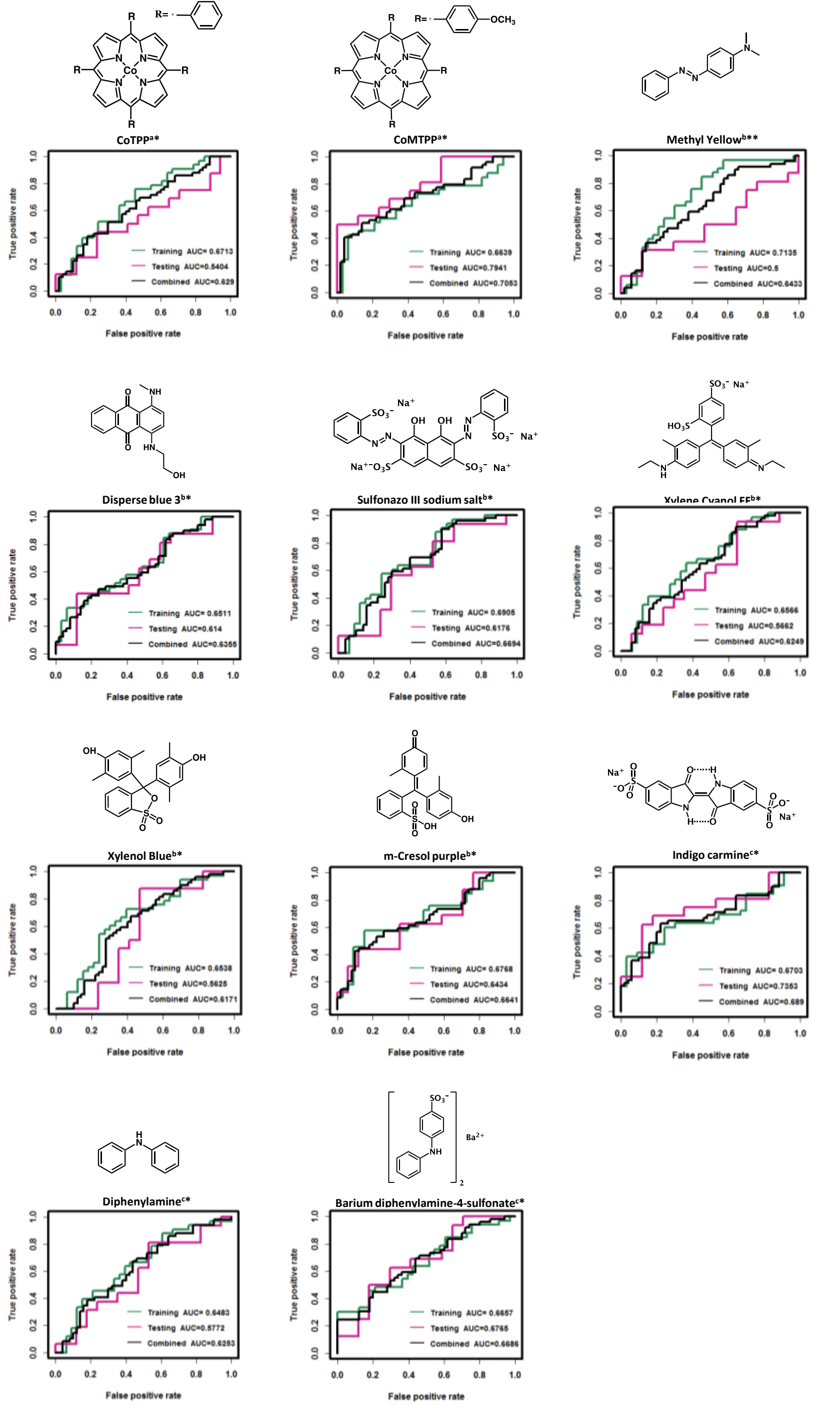

[a] Lewis acid/base dyes (metalloporphyrins); [b] Brønsted acid/base dyes; [c] Redox indicator
* 0.005≤*P* value<0.05; ** *P* value<0.005
