# Supplementary material for: Urinary Colorimetric Sensor Array and Algorithm to Distinguish Kawasaki Disease from Other Febrile Illnesses
Source: PLoS One. 2016 Feb 9;11(2):e0146733. doi: 10.1371/journal.pone.0146733 (PMC4747548; doi:10.1371/journal.pone.0146733)
Supplement: S2 Fig — (PDF) [file pone.0146733.s002.pdf]

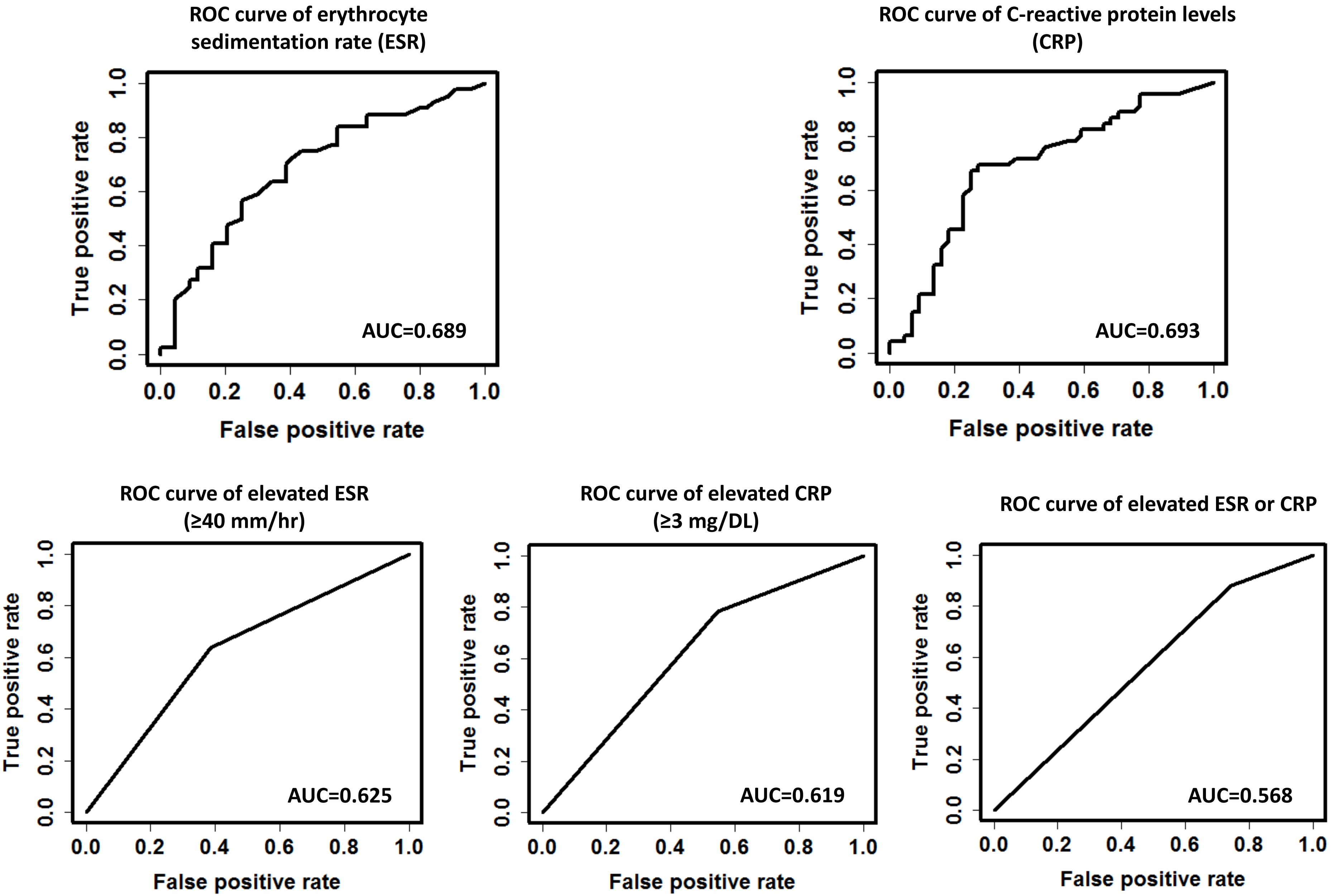
ROC curve of erythrocyte sedimentation rate (ESR)
AUC=0.689
ROC curve of C-reactive protein levels (CRP)
AUC=0.693
ROC curve of elevated ESR (≥40 mm/hr)
AUC=0.625
ROC curve of elevated CRP (≥3 mg/DL)
AUC=0.619
ROC curve of elevated ESR or CRP
AUC=0.568
True positive rate
False positive rate
0.0
0.2
0.4
0.6
0.8
1.0
