# Supplementary figures and images for: Urinary Colorimetric Sensor Array and Algorithm to Distinguish Kawasaki Disease from Other Febrile Illnesses
Source: PLoS One. 2016 Feb 9;11(2):e0146733. doi: 10.1371/journal.pone.0146733 (PMC4747548; doi:10.1371/journal.pone.0146733)

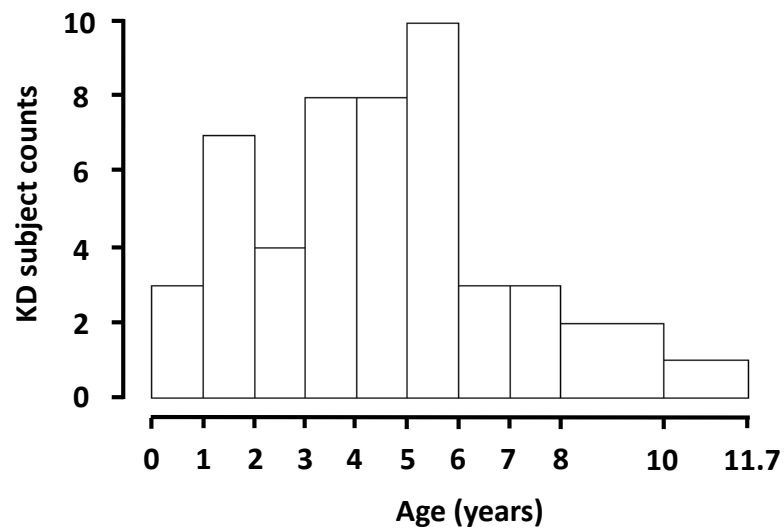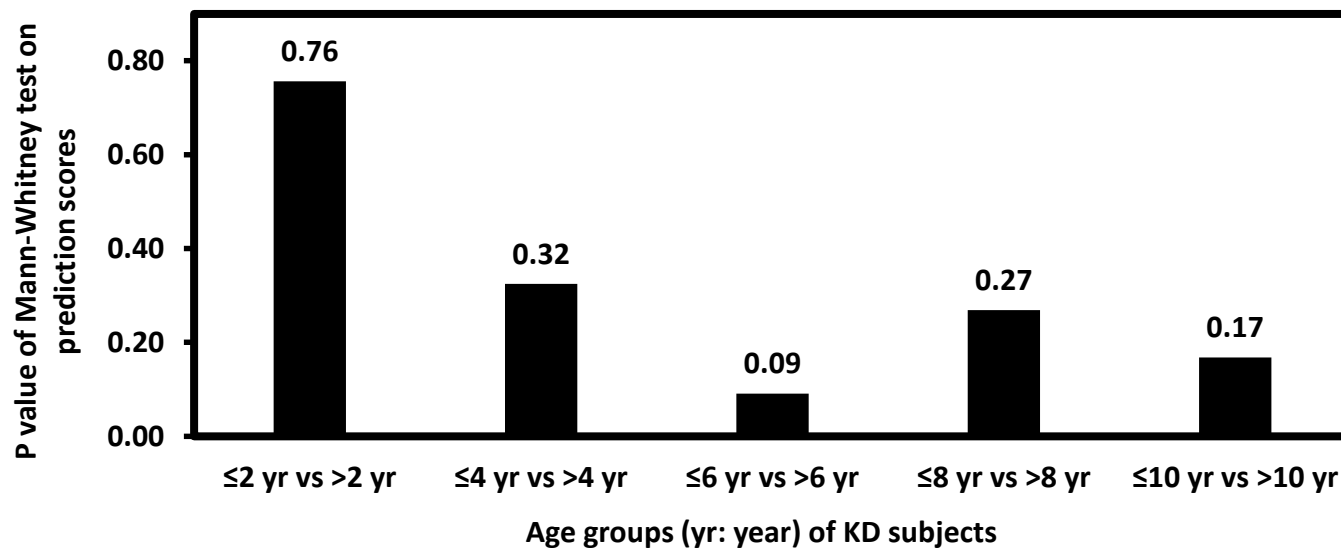

Supplement: S3 Fig — Bottom: Comparison of the algorithm-derived prediction scores be-tween younger and older groups of KD subjects. (PDF) [file pone.0146733.s003.pdf]
